# Acute port congestion and emissions exceedances as an impact of COVID-19 outcome: the case of San Pedro Bay ports

**DOI:** 10.1186/s41072-022-00126-5

**Published:** 2022-11-22

**Authors:** Luka Vukić, Kee-hung Lai

**Affiliations:** 1 Department for Management of Marine Technologies, Faculty of Maritime Studies, University of Split, Ruđera Boškovića 37, 21000 Split, Croatia; 2 Department of Logistics and Maritime Studies, Faculty of Business, The Hong Kong Polytechnic University, Hong Kong, China

**Keywords:** Air emissions, Port congestion, Anchored ships, San Pedro Bay, COVID-19

## Abstract

In the second half of 2020, the shift in consumer demand and reduction in containership capacity, as a consequence of the COVID-19 pandemic, contributed to the disruption of the global supply chains, especially on the US West Coast. This article provides an environmental view of acute maritime congestion in Los Angeles and Long Beach anchorage areas aiming to calculate air emissions of anchored ships consistently in a specific month of the year and compare the dynamics of the emission levels with previous years. The findings determine the causes of the increased environmental pollution and conclude on the preservation measures improvement. CO_2_, SO_x_, NO_x_, PM_10_, and PM_2.5_ emissions are examined in this study, considering the statistical data on port performance, productivity and competitiveness elements, ship specifications and propulsion, and emission factors of principal pollutants. Results of our mathematical calculation showed an exponential increase of air emissions generated from ships' auxiliary engines and boilers in 2021, compared with the previous periods, reaching more than 45,000 tons of pollutants emitted in November (mainly carbon dioxide). The increased port congestion and pressure upon the environment and human health also exposed the vulnerability of the intermodal chain on the landside, manifested in higher utilization of trucking services inland, contributing to the additional growth of total emissions. The environmental degradation caused by the surge in demand for products carried by container ships coincides with increased social impacts and the requirement for investments in mitigation measures for emissions to reduce the harmful effects of shipping activities.

## Introduction

Usually, the most common introduction sentence when discussing the maritime, shipping, ports, or other complementary fields of interest, would be that 90% of global trade volume is shipped by sea transport mode. This expression, predominantly used, emphasizes the magnitude and colossal impact of the shipping industry, whose presence is evident in almost each pore of everyday living. The clothes we wear, the equipment we use, or the foods we eat are all outputs of the service continuously providing resources for global consumption. But, without the consistent approach of scientists and professionals to underline something that should be a well-known fact, we would not reach a conclusion. The reasons can be found in the rudimentary perception of the public towards maritime related-businesses often taken for granted. The negative connotations towards the shipping business increase with the adverse events in the industries’ basic settings, e.g., if there are harmful market conditions, environmental pollution, congestion, logjam or accident, and disaster. Despite historical profits generated by container companies in 2021 due to the surge in freight rates, there are severe doubt and disproval concerning the role of maritime transport in disrupting the global supply chain. Against this background, the shipping industry came to the spotlight of the general interest during COVID-19 pandemics not because of its contributions on humanity and its presence in the globalization process, but its disruption concerning acute congestion created in global ports, product delivery delays, and unreliability manifested particularly on the US West Coast. The public health crisis of COVID diseases affected world trade and maritime industry proportionally. The world has only implicitly learned the fundamentals of seaborne commerce and the dependence of the western economies on the supply chain originating in Asia, where most of the manufactured goods are produced, processed, assembled, or packed. Even the container manufactured industry is supremely consolidated in China, the surge in container production was recorded in 2021, up 130% from 2020 and 62% from the record year in 2018 (Drewry 2022). The container shipping business has a central point in the globalization process and is an inherent link in the chain of dislocated production facilities and consumer demand locations. Containership is one of the technical wonders to realize the modern industry, which transports one of the most significant innovations of contemporary business, a cargo box or a container. The cargo box has promoted world trade volume and contributed to intermodality as the principal technological achievement of the twenty-first century. An enormous variety of finished and semi-finished products are predominately the content of the container, transported all over the globe to consumers not aware of the complexity of the logistical network historically shaped and established to enhance the individual features of the country's economy.

According to the International Maritime Organization (IMO) (2020), the share of shipping in global anthropogenic emissions increased from 2.76% in 2012 to 2.89% in 2018. Regarding the ship type, containerships are the most dominant source of GHG emissions in total international shipping emissions (Czermański et al. 2021). Ship emissions correlate to the sailing speed of a single ship type (Yau et al. 2012), and there is also a strong relationship between ship fuel consumption and carbon emissions (Wang and Meng 2012). According to the ship type criterion, the overall energy utilized for onboard machinery is dominant on containerships. On commercial lines, energy is primarily used for propulsion (main engine), following the remaining energy sources as the electrical power (auxiliary engine) and heat (boilers). Considering the share of four operational phases of the ship in a voyage in the greenhouse gas (GHG) emissions, it is obvious that containerships have caused the largest share of total emissions associated with periods of slow cruising, maneuvering, and berthing/anchoring (IMO 2020). It confirms the "slow steaming" initiative, with speed reduction as the single main driver for reducing shipping emissions (Psaraftis et al. 2009). In addition, the just-in-time (JIT) arrivals in port are an effective mechanism to lessen emissions as in the voyage as near the city ports (IMO 2020). The significant indicator of shipping emissions is the spending time on anchor when the total emission size is associated with port location and operations (Lighthouse 2021). When the ship is on anchorage, which is defined as one of the four-ship activity phases and corresponds to the location where ships wait for an assigned berth, the propulsion engine does not operate while the auxiliary engines are running to provide power to onboard systems (Lee et al. 2020). Poulsen and Sampson (2019) indicated the benefits of reducing the time on anchorage, which is related to the environment and business, i.e., lower costs and emissions. The study of Lighthouse (2021) indicated the most common reasons for anchoring considering the port operations processes, of which shortcomings in other segments of the transport chain and market conditions can be associated with the current trends in shipping, especially on the US West Coast. The port of Los Angeles reported a 7% increase of the total ship's CO_2_ emissions in 2020 (Starcrest 2021a), while they were 2% lower in the port of Long Beach related to the previous year (Starcrest 2021b). Due to the impact of the global pandemic, which disturbed the global shipping network, the rigid fundamentals, even in anchoring processes, shifted and atypically affected the liner shipping segment. In 2021, more than 75 containerships in San Pedro Bay were waiting for the berth to be assigned and drifting in conditions when all of the 60 anchoring spots were full (Lighthouse 2021). The increased demand for consumer goods was a primary variable that initiated the spike in anchorage calls for containerships in the Port of Los Angeles, especially throughout the second half of 2020. These consequences were determined as the main reasons to account for the increase of overall emissions in 2020 for the San Pedro Bay area (Starcrest 2021a).

This paper deals with the calculation of ship emissions on the anchorage area in the vicinity of the two ports situated in the San Pedro Bay, the Port of Los Angeles and Long Beach. Regarding the significant increase in the number of daily ships on anchor at the end of 2020, there is a need to determine and compare the ships' air pollution levels in the pre-pandemic and post-pandemic periods. For the ships' relocation in outer areas of designated anchorage in the final months of 2020, the authors included the additional emissions in the overall calculation. This research, which considers the emissions generated from ships' auxiliary engines and boilers according to the determined load factors, generates novel insights into the ships' environmental and community impact, further to supplement the conventional study on the economic disruption caused by the acute logjam in selected ports. Based on the statistical data on port performance, productivity and competitiveness elements, ship specifications and propulsion, and emission factors of principal pollutants, the authors calculated five main pollutant categories, CO_2_, SO_x_, NO_x_, PM_10_, and PM_2.5_. The environmental degradation caused by the surge in demand for container shipping coincides with increased social impact as human health issues and investments in emissions mitigation. The discontinuity of the supply chain on the US West Coast also exposed the vulnerability of the intermodal chain, where the congestion in ports was manifested with higher utilization of trucking services inland, contributing to the increase of overall emissions. The rest of this paper is organized as follows:  "Literature review" section presents a review of the most relevant articles dealing with the research problem; "Analysis of the current state and trends in LA&LB ports" section determines the current state and trends in the ports of Los Angeles and Long Beach, providing an overview of the port productivity indicators and port traffic data in the anchorage area; "Data preparation and research building" section presents the description of the problem, data preparation, and structure of the research; "Results" section presents the research results and "Discussion and conclusion" section provides discussion on the results and research problem perspectives along with the summary of main conclusions.

## Literature review

The overwide relevant literature related to this research reveals only a few recent papers dealing with the environmental implications of anchored ships and confirms the actuality of selected analysis. The authors' diverse approach for evaluating environmental impacts of port-traffic activities in the San Pedro Bay port complex is visible. Topics mainly vary between the shipping-related activities performance (berth, anchor, port performance activities), port-land activities (vehicles, road, rail), and terminal-handling equipment activities. The annual air emissions inventory (Starcrest 2021a, 2021b) is the most notable document that provides a detailed and comprehensive overview of the air quality and emissions generated from several maritime-related sources in the two ports (LA and LB). In addition to determining the emissions from ocean-going vessels, the analysis comprises the emissions from harbor crafts, cargo handling equipment, rail locomotives, and heavy-duty vehicles, with the quantification of the following exhaust emissions: diesel particulate matter (DPM), PM, NO_x_, SO_x_, hydrocarbons (HC), carbon monoxide (CO) and carbon dioxide equivalent (CO_2e_). By publishing the regular annual activity-based emissions inventory, it is possible to determine the dynamics and trends in the emission reduction strategies implementation. The analysis of ocean-going vessel emission for hoteling mode at anchorage generated at combined ports Los Angeles and Long Beach in 2020 amounted to 28.1 t of PM_10_, 25.9 t of PM_2.5_, 17.8 t of DPM, 1160.4 t of NO_x_, 64 t of SO_x_, 116.3 t of CO, 43.7 t of HC and 102,162.3 t of CO_2e_. The comparison of y–o-y dynamics of these emissions indicated that emissions from vessels at anchor almost doubled in 2020 compared to the levels recorded in 2019 (Starcrest 2021a, 2021b), as a consequence of increased demand and a growing number of vessels at anchorages. Cohan et al. (2011) examined the impact of generated pollution from roadway emissions, direct port activity, cargo handling equipment, and commercial vessels in the San Pedro Bay. Applying the selected models to determine the emission sources in cold and hot months indicated the significant impact of the port activity on pollution in-port, while the communities in the port vicinity are most sensitive to road-related emissions. Kent and Haralambides (2022) marked the most influential items leading to the supply chain disruption crisis in the US. The authors explored the primary causes that accelerated severe congestion in the ports of Los Angeles and Long Beach. They have been related to several elements which impacted the port performance indicators leading to the constraints in warehousing and trucking industry capacity. Also, the possibility of shifting the supply chain by applying the near-shoring and re-shoring strategies has been questioned. The research from Kim et al. (2011) aimed to determine the emissions reduction of yard tractors by assessing the life-cycle when shifting to electric vehicles instead of conventional diesel-powered equipment. Besides the significant decrease in the emissions generated and pollution (TTW vs. WTT emissions), the study indicated the notable impact of the container throughput in LA port on the total cargo-handling equipment emissions. Ault et al. (2009) examined the transported aerosol influence on PM concentrations in the vicinity of the Los Angeles and Long Beach ports. Measuring the concentrations of submicrometer carbonaceous and transition metal particles, aiming to quantify the environmental impact of regional transport in the San Diego region, the study reveals the significant influence of ports LA and LB and the total shipping-related activities on the air quality in California. Hu et al. (2008) similarly monitored PM emissions in the ports LA and LB, indicating that the increased in-port traffic emissions can substantially increase the potential of airborne PM and induce oxidative stress of human cells. Moretti and Neidell (2011) estimated the health effects of ozone, considering the vessel traffic and port of Los Angeles and Long Beach as variables in terms of pollution social-costs calculation. The results reveal a strong connection between respiratory-related hospitalizations and port traffic pollution. Kuo and Saphores (2015) analyzed the effects and benefits of policies and initiatives to significantly lower the GHG and air pollutants from cargo operations in the San Pedro Bay Ports (LA and LB). The authors show that besides the significant reduction of heavy-duty vehicle emissions of NOx by 80% and PM by 90% in the period 2005–2012, the success of the clean air programs strongly depends on the decrease in the port-related operations of trucks and trains outside the port complex.

## Analysis of the current state and trends in LA and LB ports

The following chapter provides an overview of the fundamental data considering the port traffic and principal port indicators to determine the current trends which affected their operational activity and generated environmental pollution.

### The overview of the port productivity indicators affected by the congestion

**Fig. 1:**
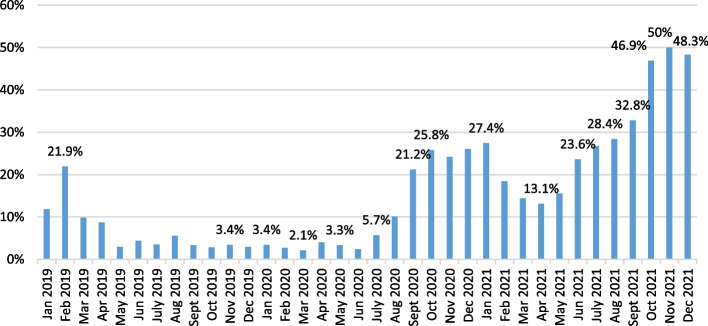
Share of containers at San Pedro Bay terminals for more than five days. *Source:* PMSA (2021)

Following the supply chain disruption crisis, many new indices measure the diversity of global economic data, e.g., the supply, container shipping efficiency, logistics pressure, supply chain pressure, trade indicators, and others. They reveal the strong commitment to resolve the logistics constraints and eradicate the uncertainties caused by the pandemic. The trans-pacific corridor has been the busiest container world route, transporting 31.2 million TEUs in 2020 or 21% of the world's container trade (UNCTAD 2021). On the western part of the shipping lane, the Port of Los Angeles (LA) has been the central gateway for international trade in the Western hemisphere. Besides the Los Angeles port, the San Pedro Bay Port Complex also includes the Port of Long Beach (LB), which acts as a diverse entity and separate department (Port of LA 2021a). These ports (LA&LB) jointly contribute to 40% of all seaborne container imports in the USA. The Port of Los Angeles handled 10.7 million TEU in 2021, an increase of 15.9% compared to the previous year, while the Port of Long Beach reported growth of 15.7% or 8.1 million TEU. The combined container handled volume of the two ports in the San Pedro Bay was 20.1 million TEU in 2021. The causes of these astonishing results are in various operational, behavioral, market, and other factors mainly influenced by the change in consumer demand related to pre-pandemic periods. The US e-commerce rose exponentially in 2020 by 32.6%, more than double the share recorded in 2019, while the projection for 2021 is set to 16.1% (S&P Global 2021). The shift in buying habits, or demands for goods, initiated the surge in demand for containerships, which were affected by imbalances and shortages of overall container movement capacity. These consequences resulted in a spike in shipping prices. The combination of high demand for goods and manufacturing supplies in the US, and short supply of containerships, caused historical port congestion in the US main import ports, Port of Los Angeles and Long Beach. There were more than 100 ships in the queue and unbelievable 23 waiting days for berth available in the Port of Los Angeles and Long Beach (Kent and Haralambides 2022). A severe bottleneck had an implication not only on the maritime component of the intermodal chain but also on disruption inland, like the issue of repositioning empty containers stuck on terminals, lack of warehouse space, short supply of chassis, and shortage of workers, especially in trucking business and warehouses, both affected by the COVID-19 pandemics (CNN 2021). Thus, the inability to receive the cargo from containerships and further to distribute the outbound containers by truck disrupted the supply chain. Overall, the waiting time of containers on terminals designated for export has increased to two weeks, a change of three to four days more than recorded in the pre-pandemic era (Klachkin 2021). Roughly 35% of the intermodal containers in LA port are handled by rail, which contains one near-dock railyard and five additional on-dock railyards for seven container terminals (Port of LA 2021b). Since the outbreak of COVID-19, these intermodal loads transported by rail decreased by over 1% of the market share compared to long-distance trucking service despite the advantages in costs and environmental competitiveness (Gross Transportation Consulting 2022). The severe congestion negatively affected the fundamental indicators of port productivity, performance, transit time, berthing time, and dwell time (Lloyd's List 2021). Average weighted dwell time, which represents the time a container spends at the terminal after completion of the unloading activity from a containership and taken off by a truck, for a laden inbound container in San Pedro Bay increased by 72.6% in November 2021 when comparing the same values recorded in November 2020. Also, by calculating the average dwell time in days for the individual business year, where the percentage reflects the share of containers held at LA&LB container terminals for more than five days, the year-on-year increase of almost 250% was accomplished in 2021 and peaked in November when the share was around 50% (PMSA 2021). Figure 1 represents the share of containers that remained at San Pedro Bay terminals for more than five days.

Contrary, the average rail dwell time was 8.6 days in 2021, having a peak in April (12.4 days). An overall downward trend in the second half of the year culminated with a dwell time of 3.5 days in November (PMSA 2021). As already mentioned, the transit and berthing time also suffered the harsh impact of port congestion. The transit time from ports located in China until container discharge in ports LA&LB rose by more than double, from the nominal 16 days (route Ningbo, Qingdao, Shanghai, Yantian, LA&LB) of sailing to more than 35 days. With a delivery time to the final customer included, the overall transit time increased more than 50% from the original 25 days. Berthing time in the ports of LA&LB also peaked in later 2021, an average of 8 to 9 days in the last quarter (Lloyd's List, 2021). The Ports of LA&LB productivity can be expressed through the import–export ratio of full and empty containers. Throughout the calendar year 2021, the ports exported three empty containers for every loaded container, which clearly illustrates the trade imbalance and terminal congestion. Contrary, almost every imported container to the ports of LA&LB was loaded (full) (Port of LA 2022; Port of LB 2022).

### Analysis of port traffic data in the anchorage area of the LA&LB ports

**Fig. 2:**
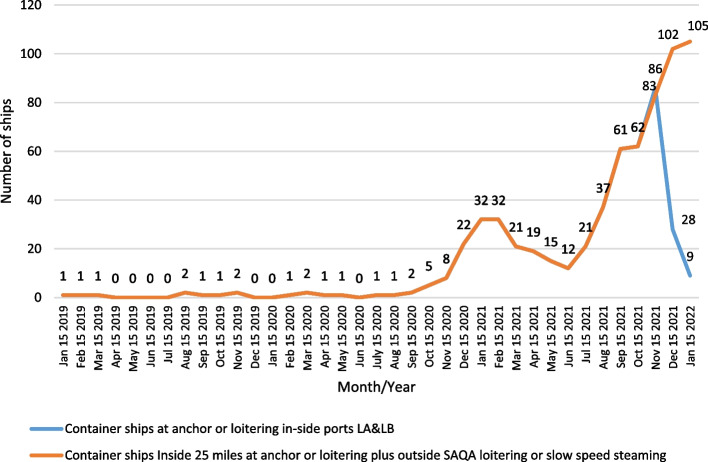
Number of containerships at anchor and loitering inside and outside SAQA of LA and LB ports. *since mid-November new methodology (counting system) *Source:* MXSOCAL (2021).

In San Pedro Bay, the overview of the issues can be expressed through the indicator of the overall number of anchored containerships, waiting for berth allocation in the ports’ areas of LA and LB by months throughout the year. According to the historical overview of vessel activity, the number of vessels at the anchorage area of Los Angeles and Long Beach ports is shown in Fig. 2. The values in December 2021 were down drastically compared to the previous month due to the new calculation method implemented for queuing and counting container ships.

**Fig. 3:**
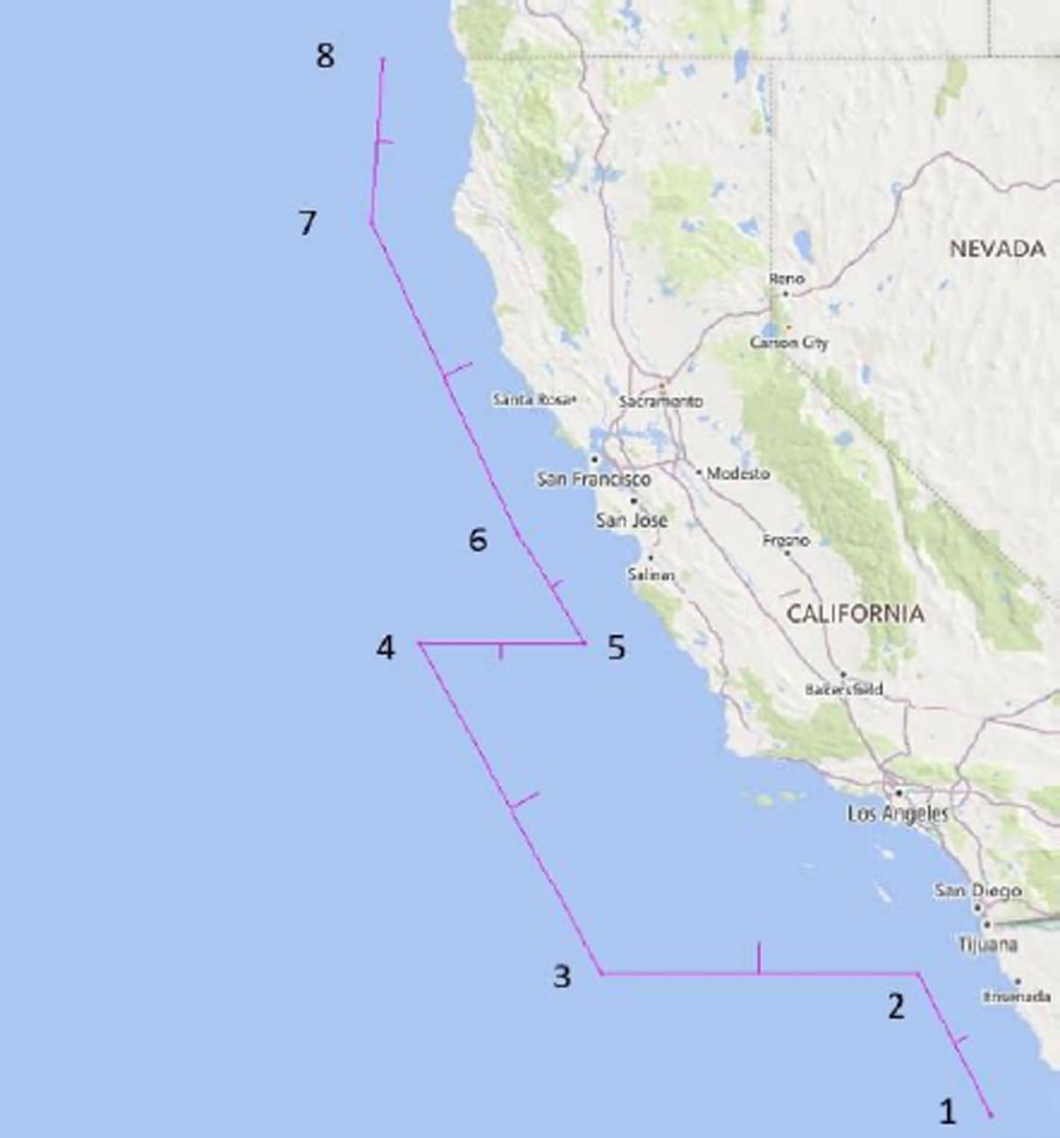
Safety and Air Quality Area (SAQA). *Source*: MXSOCAL (2021)

Figure 2 shows the port congestion expressed through the number of ships at berth in the area of ports LA and LB on a given dates, from January 15, 2019, to January 15, 2022. The values recorded throughout 2021 on a specific date show an exponential increase in the number of anchored vessels compared to the levels recorded in 2020. The difference in the number of the ships at anchor at a specific date in a month, considering the period January 2020–December 2021, increased from 1.3 to 37 times. These levels confirm the harsh impact of all the factors indicated, individually contributing to the disruption in the ports and proportionally to the overall supply chain. The vessels at berth have been excluded from the group of total anchored vessels. The number of ships on berth is a variable factor with neglected deviation, mainly for limited capacity and stable demand. In mid-November, a new model for queuing and counting container ships waiting outside the 40-mile "in port" zone was applied. It is based on the methodology which directs containerships to a specially designated Safety and Air Quality Area (SAQA) that extends 150 miles to the west of the ports and 50 miles to the north and south, waiting to be assigned a berth to unload cargo. Furthermore, besides the intention to lower the pile-up of ships close to the port entrance, the relocation of vessels aimed to decrease potential risks to maritime safety and improve air quality (MXSOCAL 2021). SAQA is shown in Fig. 3.

**Fig. 4:**
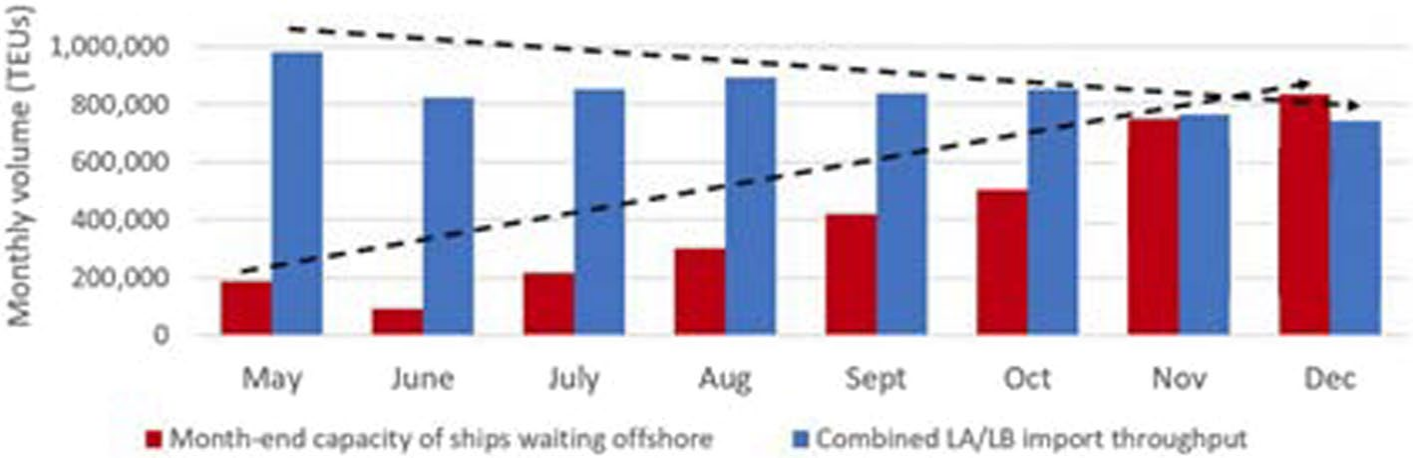
Throughput of ports LA/LB and month-end capacity of ships waiting offshore. *Source:* American Shipper (2022)

Additionally, when analyzing the ratio of combined monthly import volumes (in TEUs) to the TEU capacity of ships waiting on the anchorage and in the queue at the end of a specific month, the capacity of the ships waiting offshore exceeds the throughput of ports LA/LB in December 2021 (Fig. 4). The ships' capacity at anchor and SAQA increased by almost 650,000 TEU, while import volumes diminished by close to 240,000 TEU when comparing the values in December and May 2021.

**Fig. 5:**
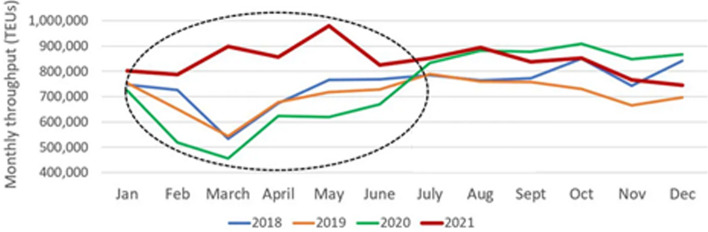
Combined monthly container imports by year (Los Angeles and Long Beach) *Source:* American Shipper (2022)

Besides the extraordinary results recorded and volumes handled in 2021, for ports LA and LB, the general productivity of the import volumes was driven by the performance achieved throughout the first half of the year, while it decreased when approaching the end of the year (Fig. 5).

**Fig. 6:**
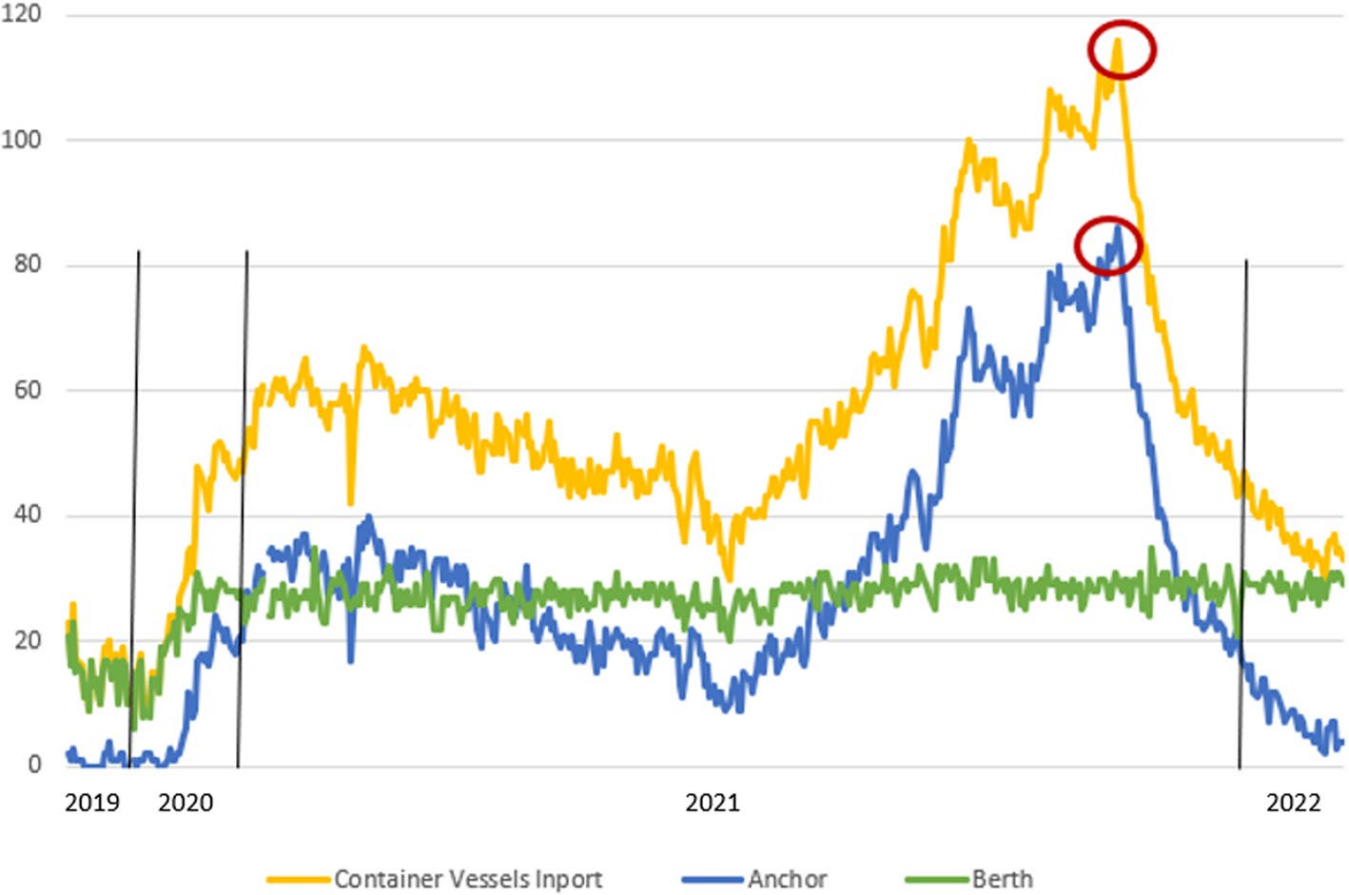
Total Container Ships inside 25 miles of Los Angeles and Long Beach Jan 1st 2019–Feb 10th 2022 *Source:* MXSOCAL (2021)

The decreasing trend of container imports in the second half of the year 2021 was an implication of the port and inland congestion, which occurred and influenced the global trade, and proportionally raised the container freight rates. Figure 6 shows the overview of containership activity inside 25 miles, considering the total container vessels in-port, ships at anchor, and berthed, from January 1st, 2019 to February 10th, 2022, for the ports in LA and LB. The conventional number of containerships at anchor in the pre-pandemic period was 0–1. The highest density of cellular ships were recorded on November 16th (2021), when 86 container vessels were at anchor or loitering inside 25 miles, which contributed to 116 reported containerships in-port, thus at berth, anchored, or loitering inside 25 miles (red circle).

Next to the new queuing model, a significant drop in all observed indicators was visible. The decline in the total number of container vessels in-port, anchored, and loitering ones inside 25 miles of LA and LB ports coincides with the increase of the total number of container vessels outside the SAQA.

**Fig. 7:**
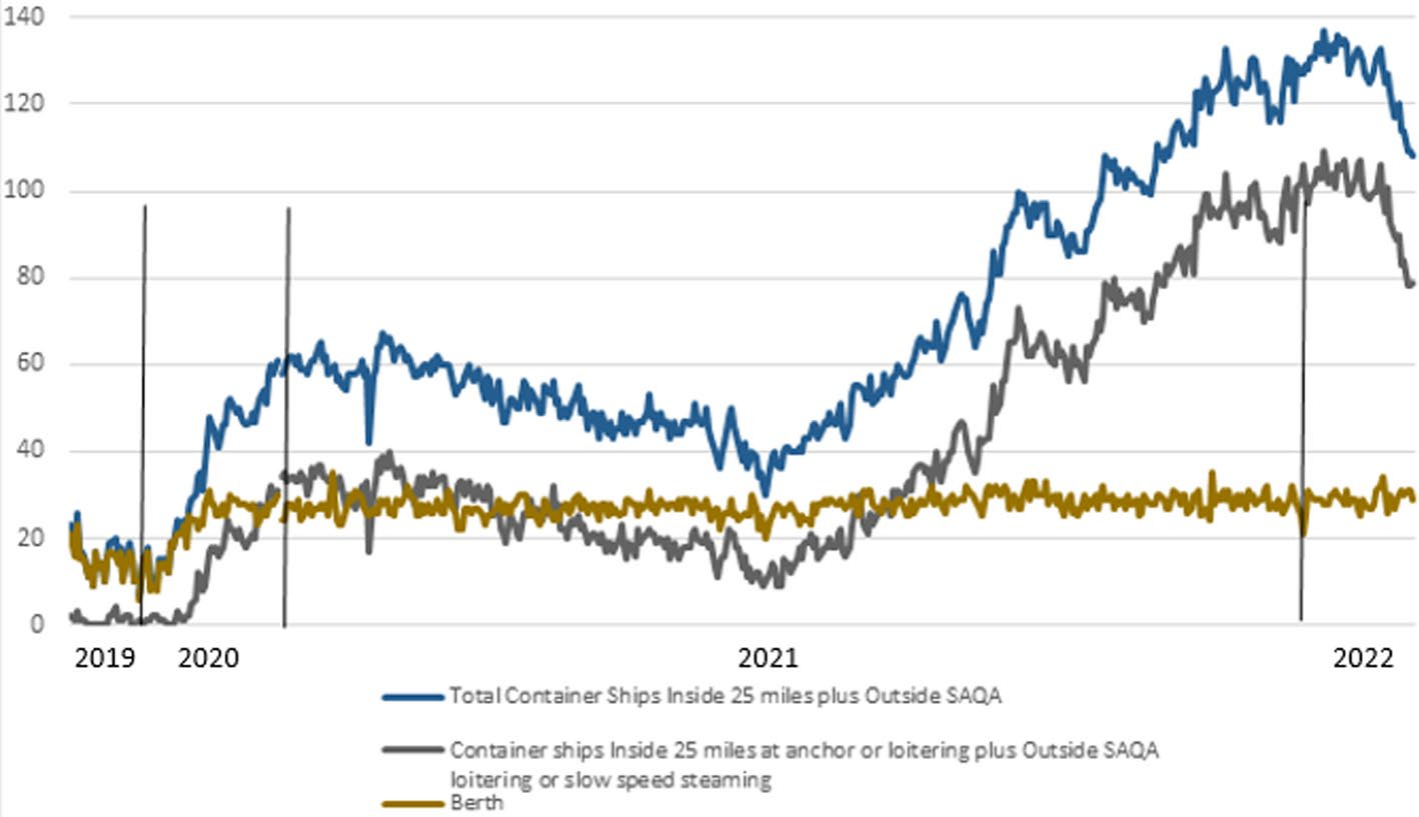
Container Ships Inside 25 miles plus Outside SAQA LA/LB 1 Jan 2019–Thu 10 Feb 2022 *Source:* MXSOCAL (2021)

Figure 7 shows the overview of containership activity inside 25 miles combined with the number of ships outside SAQA considering the total container vessels imported, anchored, and berthed ships, from January 1st, 2019–February 10th, 2022 for the ports in LA and LB.

**Fig. 8:**
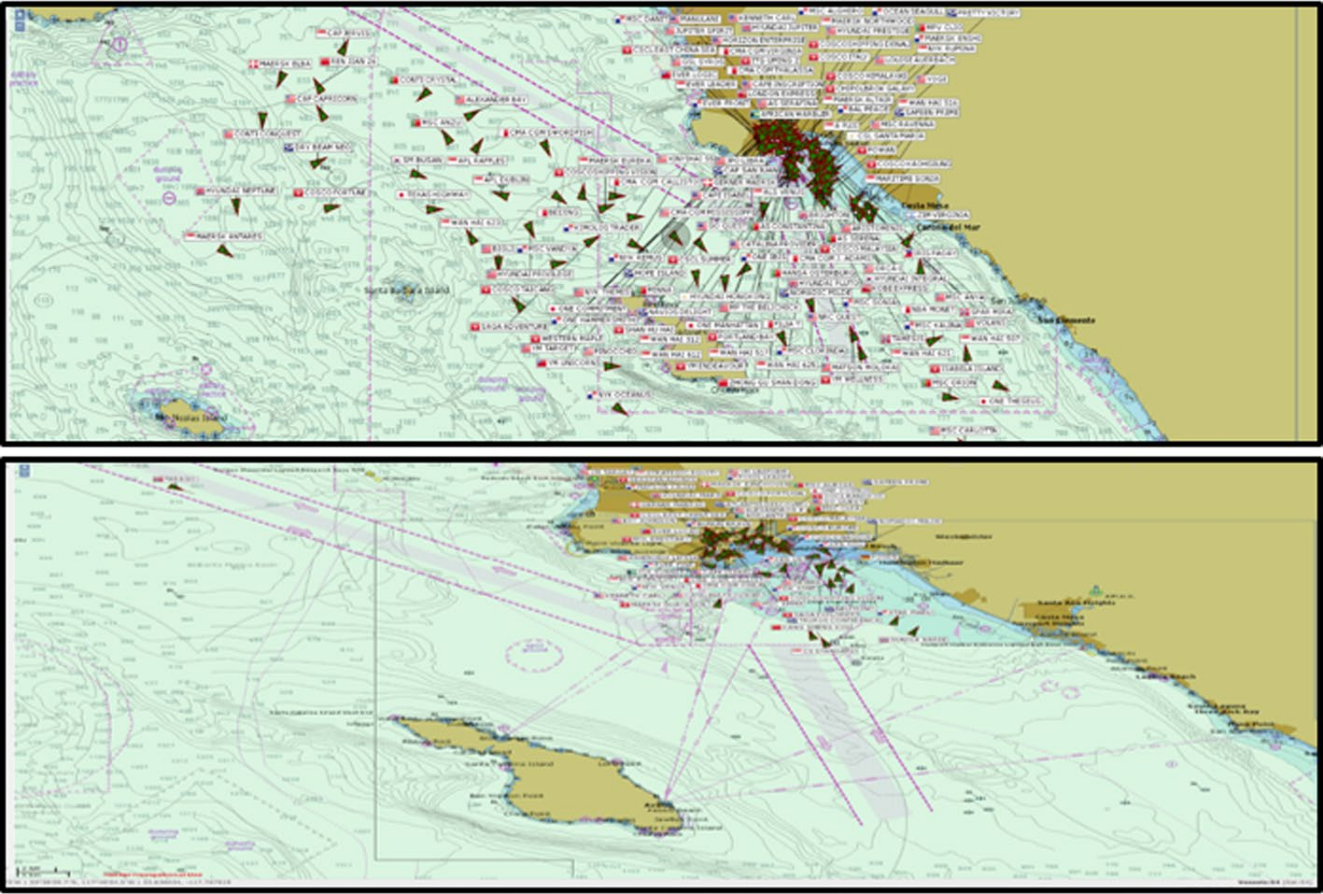
State of SAQA and shipping lanes for vessels loitering in approach to the port LA and LB on November 16th, 2021 (above) and February 3rd, 2022 (below) *Source* MXSOCAL (2021)

The grey line represents all containerships inside 25 miles at anchor or loitering and those loitering or slow speeding outside SAQA. On January 9th, there was a record high congestion, which amounted to 109 containerships. Figure 8 illustrates the effects of the new queuing system implemented by comparing the state of the SAQA area and shipping lanes on November 16th, 2021, and February 3rd, 2022. In November 2021, the images indicated 62 loitering vessels, and in February 2021, there were none of them, contributing to the overall safety and air quality.

## Data preparation and research building

According to the study performed by World Bank (2021), the LA port ended up in 328th position considering the Container Port Performance Index in 2020. The port congestion noted in San Pedro Bay during 2021 disrupted the supply chain in its operational efficiency and economic contribution to the national economy. It also influenced the increase of environmental pollution with air emissions as the main component. It was primarily a consequence of the increased number of anchored ships near the ports LA&LB and along the SAQA. The ships' auxiliary engines ensured the electricity for their equipment, and along with auxiliary boilers, they are primary sources of pollution from ships at anchor (Starcrest 2021a). The boilers probably operated during the entire voyage, thus covering all the operational phases, maneuvering, hoteling, and anchorage.

Two separated calculation models for the vessels' air emissions within the anchorage area of the LA and LB ports were used, one considering the IMO (2020) methodology, which divides the calculation on energy-based (1) and fuel-based (2) emission factors, and the other related to methodology included in Starcrest (2021c) (3). The calculations are determined according to the most commonly used methodology of emission factors computing for a specific group of pollutants. The energy-based computing of pollutant emissions comprises the power output and emissions factors in g/kWh, and the fuel-based calculation considers hourly fuel consumption (in tons) and emission factors (in gpollutant/gfuel) (IMO 2020). On the other hand, the emissions by mode in Starcrest (2021c) are calculated by taking into consideration energy, determined by load (in kW), activity (in h), and multiplied by emission factors (in g/kWh), a fuel correction factor (if applicable) and control factor (if applicable). The two diverse methods utilized in air emission calculation enable us to examine the credibility of calculated data and accompanied deviations. It was also the confirmation of the functionality of the two individual calculation algorithms.

The calculation of air emissions of vessels at anchorage considering the energy-based method can be expressed as:

1 $${EM}_{i}={{EF}_{i}*W}_{i}$$

where EM_i_ is the total emissions amount of a propulsion mode i, EF_i_ is the emission factor of a propulsion mode i, and W_i_ is the propulsion power.

Contrary, the fuel-based air emissions of selected pollutants are calculated as follows:

2 $${EM}_{i}={{FC}_{i}*EF}_{i}$$

where EM_i_ is the total amount of a propulsion mode i, FC_i_ is the fuel consumption of a propulsion mode i, and EF_i_ is the emission factor of a propulsion mode i. The fuel consumption from Eq. (2) is calculated as:

2.1 $${FC}_{ae/bo}={{SFC}_{ae/bo}*W}_{ae/bo}$$

where FC _ae/bo_ is the fuel consumption of the auxiliary engine or boiler, SFC _ae/bo_ is the specific fuel consumption of the auxiliary engine or boiler, and W _ae/bo_ is the propulsion power of the auxiliary engine or boiler.

The total air emissions of ships at anchor, according to the second methodology retrieved from Starcrest (2021c) can be expressed through the following relation:

3 $${EM}_{t}={{E}_{t}*EF}_{t}*FCF*CF$$

where EM_t_ is the total amount of a propulsion mode t, E_t_ is the energy released of a propulsion mode i, EF_t_ is an emission factor of a propulsion mode i, FCF is fuel correction factor and CF are the control factor. According to the IMO (2018), it is assumed that the ship’s AE and boiler emissions are not dependent on the load, which excludes the correction by CFL. The energy parameter can be expressed as follows:

3.1 $${E}_{t}={{P}_{t}*A}_{t}$$

where E_t_ is the total amount of energy released of a propulsion mode t, P_t_ is the power generated of a propulsion mode t (according to the designated load factor), and A_t_ is the length of the activity of a propulsion mode i.

For this research, the load factor (LF) was set at 80%, according to the data retrieved directly from the ships positioned at anchorage. Table 1 shows the emission factors determined according to the IMO (2018). The CO_2_ and SO_x_ emission factors are usually calculated as fuel-based and expressed in g/g fuel, while the remaining pollutants NO_x_, PM_10_, and PM_2.5_ are mostly output of released energy in g/kWh. It should be noted that the authors assumed the use of marine diesel oil (MDO) as combustion fuel for all the vessels at anchor, as for the inclusion of the US west coast in the emission control area (ECA).

**Table 1:** Emission factor unit values of selected pollutants. *Source:* IMO (2018, 2020)

Fuel type	Emission factor (g/g fuel)		Emission factor (g/ kWh)					
	CO_2_*	SO_x_ (in 2018)	NO_x_ (Tier 0) **		PM_10_***		PM_2.5_	
MDO	3.206	0.0014	AE	Boiler	AE	Boiler	AE	Boiler
			11.2	2.1	0.18	0.14	0.1656	0.1288

^*^IMO 2018

^**^Used as for the lack of data in IMO (2020)

^***^Generation III engines considered and year 2018

Additionally, the authors assumed that all the container vessels, which sailed the San Pedro Bay area, were built before 2001, with the SFOC of auxiliary engine for MDO engine type of 185 g/kWh, and 320 g/kWh for auxiliary boilers (IMO 2020). The average container auxiliary engine and boiler load defaults for anchorage hoteling mode during 2020 in LA&LB ports are provided in Table 2.

**Table 2:** Average auxiliary engine and boiler load defaults for ships at anchorage in the Port of Los Angeles (2020) and the Port of Long Beach. *Source*: Starcrest 2021a; 2021b

Capacity (in TEU)	Port of Los Angeles		Port of Long Beach	
	Auxiliary engine (in kW)	Auxiliary boiler (in kW)	Auxiliary engine (in kW)	Auxiliary boiler (in kW)
1000	1000	230	1000	376
2000	528	441	1012	180
3000	559	517	713	361
4000	1056	456	704	487
5000	900	601	982	477
6000	1266	612	1274	757
7000	826	594	/	/
8000	1052	588	1484	554
9000	1174	722	1114	513
10,000	1181	656	1028	598
11,000	980	516	1009	463
12,000	1724	687	1776	677
13,000	1319	558	1165	594
14,000	1155	532	1224	696
15,000	1100	402	1100	402
16,000	1271	470	1050	525
17,000	1441	537	/	/
19,000	1475	783	1100	848
23,000	1549	822	1155	890

Based on the data indicated in Table 2, the authors determined the average unique value of the auxiliary engine and boiler power for both ports for the overall emissions calculation. The average auxiliary engine power was 1123 kW, while the average boiler power was 559 kW.

Considering the added methodology applied to calculate air emissions from anchored ships in ports LA&LB, the authors took over the pollutant emission factors for auxiliary engines from Starcrest (2021a, 2021b). According to the California Air Resources Board (CARB) regulation requirements in the port area, the EF was based on the 0.1% sulfur MGO fuel. The EF for five main pollutant categories (CO_2_, SO_x_, NO_x_, PM_10_, and PM_2.5_) are shown in Table 3. The AE type and emission factors of individual pollutants applied in the research were based on the percent of ship activity and arrivals. The author determined the most frequent ME Tier standard for vessels arriving in the port according to the main engine features. It was necessary for the lack of relevant data on the correlation between auxiliary engine tier standard and containership arrivals at the LA and LB ports.

**Table 3:** Emission factors of selected pollutant categories for auxiliary engines (g/kWh). *Source*: Starcrest 2021a; 2021b

0.1% S MGO	IMO TIER	PM10	PM2.5	NOx	SOx	CO2
Medium speed auxiliary	Tier I	0.189	0.174	12.2	0.424	696
Medium speed auxiliary	Tier II	0.189	0.174	10.5	0.424	696
High speed auxiliary	Tier I	0.189	0.174	9.8	0.424	696
High speed auxiliary	Tier II	0.189	0.174	7.7	0.424	696
Average		0.189	0.174	10.05	0.424	696

Besides the AE used as a primary source of electricity on-board, the ship also uses one or more auxiliary boilers for fuel heating and producing hot water and steam. These boilers are a secondary source of emission of vessels at the anchorage area. Emission factors for auxiliary boilers are shown in Table 4. The average SFOC value of the boiler is 290 g/kWh which relates to distillate fuel (Starcrest 2021b).

**Table 4:** Emission factors for auxiliary boilers (g/kWh). *Source*: Starcrest 2021a; 2021b

0.1% S MGO	PM10	PM2.5	NOx	SOx	CO2
Steam boiler	0.202	0.186	2.0	0.587	962

**Fig. 9:**
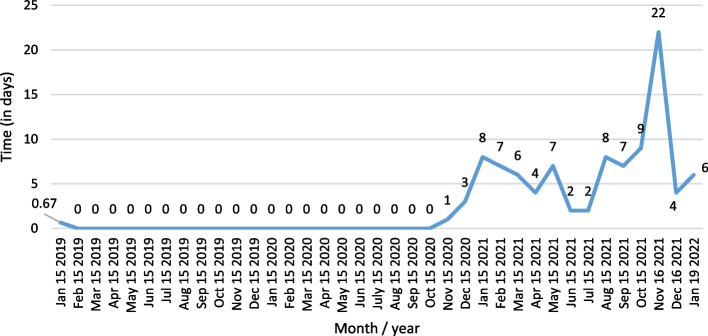
Average time of ships at anchor at Port LA (in days). *Source:* Port of Los Angeles (2021c)

According to statistical data of total containership activity in the port LA from January 15th, 2020 to January 19th, 2021, their average time at anchor is shown in Fig. 9. The time parameter of ships at anchor on a given date was used in both calculations and applied for all containerships in the San Pedro Bay. It was necessary for the limited data on the anchorage time at port Long Beach.

**Fig. 10:**
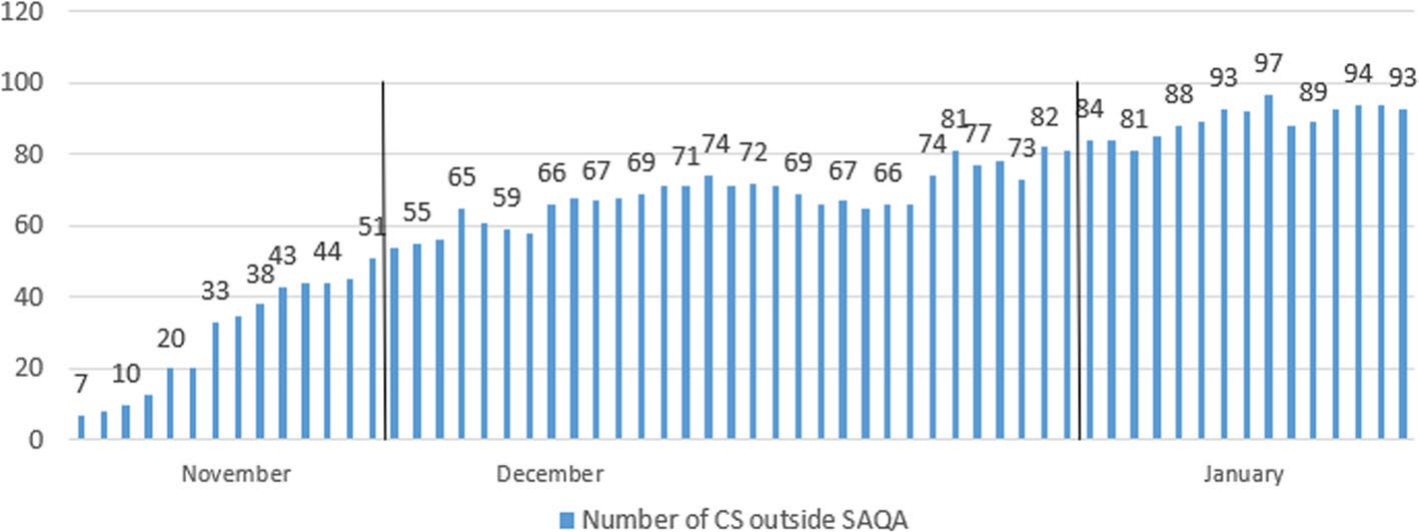
Daily number of containerships outside SAQA in 2021 (selected dates). *Source:* MXSOCAL (2021)

CARB (2008) determined the average anchorage times of 11 h per visit in the analyzed area. Due to the new queuing system introduced in mid-November, the vessels outside SAQA should also be considered when determining the increase of air pollution in the surrounding or vicinity of the port area. Figure 10 represents the daily number of containerships outside the SAQA on selected dates. The authors consider all the vessels being anchored outside SAQA, even though these vessels are steaming slow-speeding or loitering. These vessels would have been anchored inside a 40-mile port zone before the SAQA had been established.

At the end of the analysis of primary research objective, a simulation was presented to determine the extent of pollution in the case of a potential representative container ship voyage on the Shanghai-Los Angeles route and crossing the Pacific Ocean at lower speeds while avoiding waiting times at the destination port. EcoTransIT World (2022) software was used for the simulation. The same tool was also used to estimate the contribution of truck traffic to total emissions at the port of LA / LB.

## Results

**Fig. 11:**
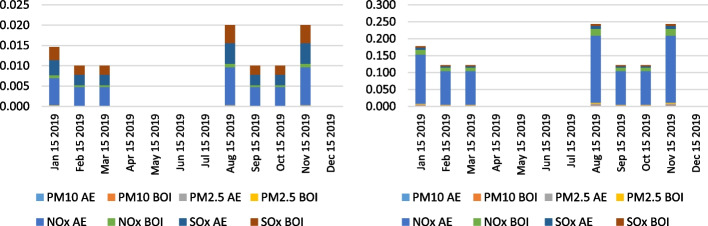
Health-related emission calculation of ships at anchorage area of the ports LA&LB based on energy and fuel-based methodology (left) and Starcrest (right) excluding CO_2_ in 2019 (in the average waiting time by months)

**Fig. 12:**
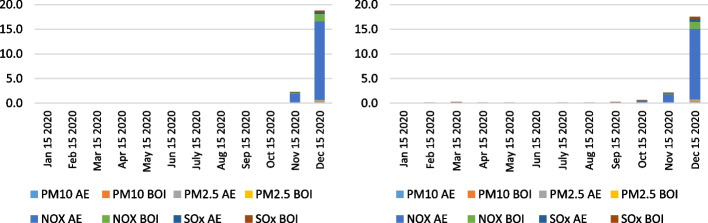
Health-related emission calculation of ships at anchorage area of the ports LA&LB based on energy and fuel-based methodology (left) and Starcrest (right) excluding CO_2_ in 2020 (in the average waiting time by months)

**Fig. 13:**
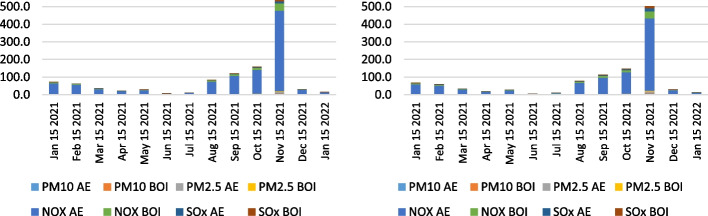
Health-related emission calculation of ships at anchorage area of the ports LA&LB based on energy and fuel-based methodology (left) and Starcrest (right) excluding CO_2_ in 2021 (in the average waiting time by months)

The empirical calculation of air emissions in the anchorage area of the LA and LB ports for the period 2019–2021 is presented in the following figures. As for the complexity and comprehensiveness of the data generated, the research results were presented as health-related emissions, measuring the overall emissions of NO_x_, SO_x_, PM_10_, and PM_2.5_ pollutants as an output of the vessels' auxiliary engines and boilers on anchorage and CO_2_ emissions. The CO_2_ emission presents the most influential and, ultimately, the benchmark of total ship emissions generated in the specific area. The results were determined by the two individual calculation methodologies performed to examine the differences and verify the results. Figures 11, 12 and 13 show the overall health-related emission calculation excluding CO_2_ on the anchorage area of the ports LA&LB, based on selected methodologies from 2019 to 2021 with a monthly overview.

The analysis of the results of health-related emission calculation in the period between 2019 and 2021 reflects the exponential increase of all examined pollutants. Also, marginal statistical differences in total emissions generated are visible depending on the two utilized methodologies. In 2019, the highest level of health-related emissions was in November (and August), when the total emissions without waiting time were 0.02, respectively 0.25 tons, according to the two applied methodologies. The total emissions generated, excluding CO_2_ emissions, in the same month in 2020 increased to 2.1 and 2.3 tons in a day of waiting time, while the highest amount of overall health-related pollutants was in December, varying from 17.5 to 18.8 in three days. Finally, the most port congestion environmental impact was in 2021, when the emissions amounted from 502.5 to 538.8 tons in November, in 22 days of waiting time, which fell in December by approximately 94% and amounted from 29.7 to 31.9 tons in four days. The decline in emissions is primarily a consequence of the novel queuing model applied, which relocated the anchored and loitering vessels from the vicinity of the port to the outer area located 150 miles from the container terminals.

**Fig. 14:**
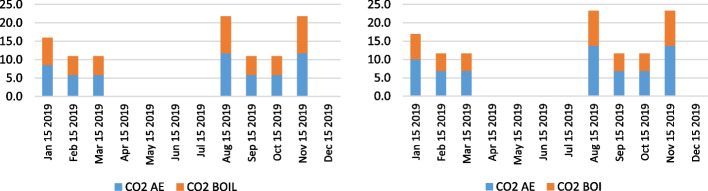
CO2 emission calculation of ships at anchorage area of the ports LA&LB based on energy and fuel-based methodology (left) and Starcrest (right) in 2019 (in the average waiting time by months)

**Fig. 15:**
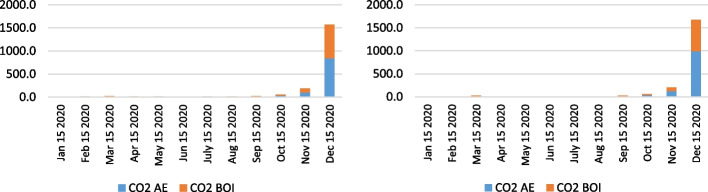
CO2 emission calculation of ships at anchorage area of the ports LA&LB based on energy and fuel-based methodology (left) and Starcrest (right) in 2020 (in the average waiting time by months)

**Fig. 16:**
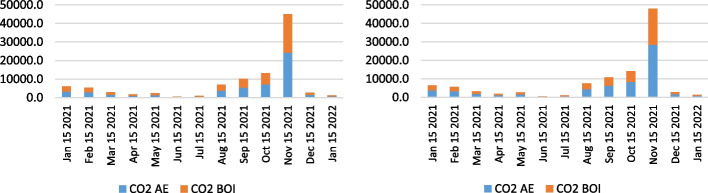
CO2 emission calculation of ships at anchorage area of the ports LA&LB based on energy and fuel-based methodology (left) and Starcrest (right) in 2021 (in the average waiting time by months)

Besides the analysis of the health-related emissions, namely NO_x_, SO_x_, PM_10_, and PM_2.5_, the authors examined the CO_2_ emissions as a fundamental pollutant having the largest share of total emissions from ships, considering the combustion of auxiliary engines and boilers. Figures 14, 15 and 16 indicate the CO_2_ emission calculation of the overall state on the anchorage area of the ports LA&LB, based on selected methodologies, from 2019 to 2021 with a monthly overview.

The results of the air emission analysis from vessels at anchor confirm the premise of the fundamental influence of CO_2_ on the environmental pollution on the examined anchorage area of ports LA and LB. From the initial emissions generated in November 2019 varying from 21.8 to 23.2 tons, when there were no waiting days, the total CO_2_ emissions in 2020 surged to 190.4 and 202.7 tons in a day of the waiting time. Also, in December 2020, considering the exponential increase of overall ships at anchor and waiting time of three days, the total CO_2_ emissions increased to values of 1570.8 and 1671.9 tons depending on the applied calculation model. With the continuation of supply chain disruptive effects, having influenced the growth of ships at the anchorage area of ports LA and LB, which peaked in November 2021, the total CO_2_ emissions peaked at astonishing levels between 45,028.5 and 47,927.8 tons in 22 day period of waiting time. However, the carbon dioxide emissions from ships' auxiliary engines and boilers decreased to 2665.5 and 2837.2 tons in December 2021 in four waiting days.

**Fig. 17:**
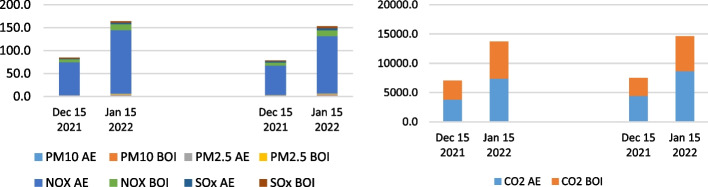
Health-related emission (left) and CO_2_ emission (right) calculation of ships outside SAQA based on selected methodologies in 2021 (in the average waiting time by months)

When analyzing the total air emissions of ships at anchor near the ports of LA and LB in the examined period, the values in December 2021 and January 2022 need corrections by adding the ships' emissions that were being relocated outside the designated anchorage area. The calculation was performed after the assumption that all the ships were at anchor even though some moved around. The calculation of the lower limit of air emissions was selected as a criterion. Total air emissions were calculated with the accepted methodological propositions assuming the determined average time of ships at anchor as the overall duration of activity of the vessels at SAQA. Figure 17 represents the entire health-related and CO_2_ ships' emissions outside SAQA based on two applied methodologies, excluding CO_2_ emissions in December 2021 and January 2022.

The calculated air emissions from vessels based on the selected methodologies reflect the severe environmental impact of port congestion and overall supply chain issues considering the area of San Pedro Bay, thus in the vicinity of the ports LA and LB. Considering the total air emissions of vessels at anchorage and outside SAQA as a reference point for the analysis, the derivations in values for each year in a selected month are visible. Table 5 shows the differences in the total air emissions of anchored ships and those outside SAQA from 2019 to 2021 in the same specified month. The calculated values are based on IMO (2020) methodology.

**Table 5:** Total air emissions of anchored ships and ships outside SAQA from 2019 to 2021 in the average waiting time (wt) in December (in tons)

December (wt)	PM_10_	PM_2.5_	NO_x_	SO_x_	CO_2_	Total
2019 (0 days)	0.0	0.0	0.0	0.0	0.0	0.0
2020 (3 days)	0.4	0.3	17.4	0.7	1,570.8	1,589.6
2021 (4 days)	2.1	2.1	107.7	4.2	9,710.2	9,826.3

The overall air emissions from vessels in the vicinity of the Los Angeles and Long Beach ports increased in December 2021 more than six times when comparing the same values with the previous year. Also, as indicated in Table 5, there were no significant emissions generated from anchored ships in 2019.

To determine the potential savings in anchorage waiting time and thus emissions, the authors applied simulations of a containership on the Shanghai-Los Angeles route as it crossed the Pacific at lower speeds. Potentially, total pollution could be lower if transit time was longer and queuing time was shorter. The simulation was performed using EcotransIT World software, considering the following data. The average speed of container ships has varied between 13.9 and 15.3 knots over the last decade (American Shipper, 2021). The average transit time on the Shanghai—Los Angeles route was 16–18 days and increased to 19–36 days during the pandemic (The Maritime Executive 2021). By 2001, the largest container ship had a capacity of 8400 TEU (Sánchez et al. 2020). These data were important for the choice of the representative container ship and the speed mode in the simulation. The date chosen was December 15, 2021, since most of the data were available for that exact date. In addition, the traffic congestion in the port LA /LB was neither the highest nor the lowest in this month. On that day, a total of 130 container ships were queued in different areas outside the port LA /LB (Figs. 2 and 7) with a total capacity of about 800,000 TEU (Fig. 4) or 6000 TEU per ship. The average time at anchor was four days (Fig. 9). CO_2_ emissions during this period were 9710.2 t and health-related emissions were 116.1 t (Table 5). All these data were used for the calculation. Table 6 shows the representative emissions of containership depending on different speed modes on the hypothetic route Shanghai—Los Angeles.

**Table 6:** Dependence of emissions on the speed mode of the containership on the Shanghai—Los Angeles route. *Source*: EcoTransIT World (2022)

Speed	Standard	Half	Low	Full
Reduction (%)	37	50	70	0
Transit time (approx. days)	17	21	35	11
Emissions (TTW* in tons)				
CO_2_	3,937	3,047	2,174	8,048
SO_2_	0.097	0.044	0.03	0.121
NO_2_	0.095	0.07	0.042	0.206
PM_10_	0.008	0.006	0.004	0.017
NMHC**	0.003	0.002	0.001	0.007

*TTW tank to wheel

**non-methane hydrocarbon

Containership: CC Transpacific trade (17 k TEU), LF: 80%, Standard (average)

Speed: 14.37 knots, Distance: 10,550.97 km, Cargo weight: 6,000 TEU (t/TEU: 10)

Theoretically, it is clear that in half-speed mode, CO_2_ emissions could be reduced by about 890 t (23%) and health-related emissions by 0.081 t (40%) per trip. Considering the 4-day longer voyage, the waiting time could be eliminated for the analyzed month (December 15th 2021), reducing emissions by an additional 74.7 t of CO2 and 0.9 t of health-related gasses per ship that would be released over the next four days before reaching the port LA /LB. Along the way, it would achieve significant fuel cost savings. The results in Table 6 also show an extreme rate of air emissions in full-speed mode (more than double that in standard mode).

Assuming that all cargo is handled by truck transport (drayage) at the destination port, the simulation provided estimates of a container-truck transport emissions per vessel. The length of the road approach to the port of LA /LB estimated by the software is 45.16 km. The obtained data can be considered as maximum values (Table 7). Actual data on the volume of cargo transported by road or rail or retained at the port for transfer to feeders may be the subject of a separate study.

**Table 7:** Truck emissions in LA/LB port (drayage per containership). *Source*: EcoTransIT World (2022)

Emissions	TTW* in tons
CO_2_	141.33
SO_2_	0.003
NO_2_	0.382
PM_10_	0.006
NMHC**	0.006

*TTW tank to wheel

**non-methane hydrocarbon

Truck (26–40 t, diesel, EURO 5, LF: 95.77%, ETF: 20%),

Distance: 45.16 km, Cargo weight: 6,000 TEU (t/TEU: 10)

The drayage of containers from the simulated containership exclusively by truck leads to additional emissions of 141 tons of CO_2_ and 0.4 tons of harmful gasses in the port area LA /LB.

## Discussion and conclusion

The shift in consumer demand as a consequence of the effects of COVID-19 on the global economic system initiates severe congestion in US ports, especially on the western coast. The Los Angeles and Longs Beach ports are the crucial nodes in the US economy, contributing to nearly half of the overall container imports and additional complexity for achieving the sustainability of the contemporary logistical network. The increase in trade and demand for various products and simultaneous vessel capacity, combined with congestion in both foreland and inland, resulted in a significant enlargement in air emissions in the whole San Pedro Bay area. The ship on anchor affects the local air quality and everyday living in the coastal zone (Clear Seas 2022). Even though the anchored ships emit less as the ships' main engine is turned off, a large number of vessels and long waiting times produce significant GHG (CO_2_) and health-related emissions (NO_x_, SO_x_, PM_10_, and PM_2.5_), and proportionally influence the socio-ecological component of the sustainability concept.

The comprehensiveness of statistical data on total traffic and other port indicators in LA and LB ports determines the need to analyze the emissions generated in a single month between 2019 and 2021. These criteria enabled the comparison of the results and indicated differences throughout the observed period. Also, considering the different methodological setup of calculation methods, two models were used to examine the total tons of emitted pollutants from ships in the vicinity of ports LA&LB, comprising the anchorage area and SAQA. The calculation of air emissions, based on engine specifics, emission factors of pollutants, and load factors, were backed-up with the data on the change in essential port indicators, which showed lower productivity, efficiency, and reliability. Conclusions were drawn based on the retrieved data, selected methodology, and determined research goals.

Depending on the chosen calculation algorithms, the results revealed the significant and, rather worrying, exponential increase in air emissions of examined pollutants in the anchorage and outside SAQA area of the two busiest US ports in selected periods. During 2019, on selected days, no emission from ships was recorded at anchorage, as also in the whole pre-pandemic period. In the first half of 2020, the container vessels were idled due to the low consumer demand and decreased freight rates. There was also the qualitative shift in consumer demand in the latter months when the purchasing of services was converted with product acquisition. Everything resulted in a higher call for intermediaries, thus for container capacities. During the last months of 2020, these circumstances coincided with higher emissions and initiated added rise of congestion on anchorages of ports LA and LB. The overall anchored ship emissions escalated throughout 2021, peaking in November when 86 ships spent an average of 22 days and emitted more than 45,000 tons of air pollutants in that period, differing on the calculation methodology assumed. The health-related emissions contributed to about 1% of total emissions generated, while the share GHG emissions were close to 99%. The overall ship emissions generated in 2020 were 0.5% of those recorded a year after. This research results confirm the dominant impact of CO_2_ emissions in maritime transport. We noted the difference between the results of the two calculation methods applied of nearly 10%. All the scenarios indicate severe ship emissions of nitrogen dioxide, sulfur dioxide, fine particulate matter (PM_2.5_), and other pollutants which create smog and ozone, especially spreading downwind in more populated areas.

**Fig. 18:**
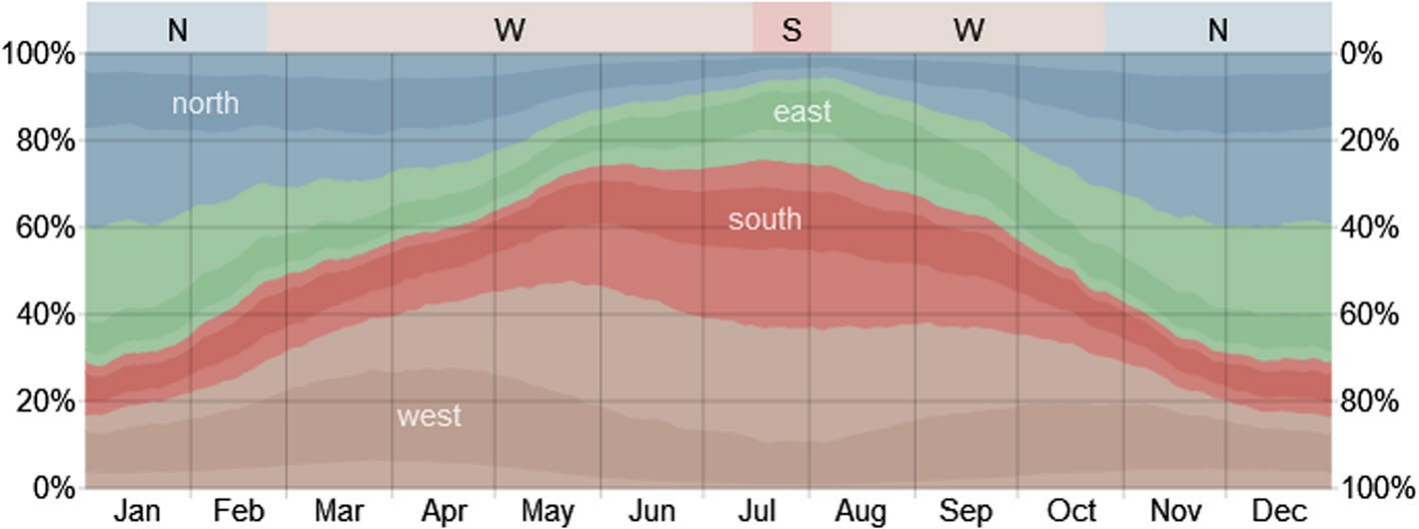
Annual wind direction in the vicinity of the ports LA&LB. *Source:* Weather Spark (2021)

The introduction of SAQA relocated the ships and simultaneously generated emissions far away from the port, changing the pollution-affected zone, but without eliminating the total emissions, thus most of the harmful consequences. For this research, the authors assumed that all anchored ships, including those outside SAQA, stayed the whole time at the place, but they had to activate their main engines or move to deeper water to ride out inclement weather. This action enables the calculation of the lower limits of anchored ships' air emissions. Based on Fig. 18, which shows the usual wind direction in the San Pedro Bay area, the period of high urban area contamination from vessels on anchor, loitering, inside and outside SAQA is determined.

As the western and southern winds predominate for more than half of the year (April–September), the pollution generated on the sea is propagated inland, thus contributing to the socio-ecological degradation. Throughout the remaining part of the year, the wind disperses the harmful gases and particles within the populated areas, influences the air and water quality, and raises concerns on climate change issues. These weather conditions are challenging and hard to predict, especially the wind direction during the lengthy period. Research by Goldberg et al. (2020) showed that the wind direction in the Los Angeles area could change NO_2_ emission concentrations by 80 percent. Lind et al. (2022) indicated more than 100 tons of pollution per day from idle ships situated in the Ports of Los Angeles and Long Beach. Converting that to on-road vehicles corresponds to pollution of more than 6 million cars. In October 2021, the estimations of CARB (2021) provided insights into the increased congestion in San Pedro Bay. They noted a surplus of 20 t/day of NO_x_ and 0.5 t/day of PM emission from anchored containerships concerning the pre-pandemic levels. These numbers are not considering all the vessels, especially those relocated outside the SAQA, but preferably provide the magnitude and implications of the acute congestion on air quality and public health.

The spillover of the effects in the seaside part of the two ports consequently was transferred in the hinterland, at the surface of the terminal, terminal gates, and access roads which created additional pollution-affected areas besides the anchorages of the two ports. The air quality in the Ports of Los Angeles and Long Beach was also the issue of NASA's Earth Observatory, which indicated the elevated concentrations of NO_2_ in the last quarter of 2021 (NASA 2021). The air emissions from trucks when loading, discharging, or performing a dual transaction were considered in the simulation and pointed to additional high pollution. The long waiting times, chassis deficit, lack of warehousing space have raised the base level of pollutant concentration. Cohan et al. (2011) empirically showed that roadway emissions are the most significant sources of local pollution, and port emissions significantly contribute to it within 6 km of the ports. This ascertainment confirms the significance of the emissions generated from the port and related activities. The build-up of empty containers locked up during pandemics in San Pedro Bay peaked during 2021, with more than 3.5 million empty containers only from the transpacific route (Sea Intelligence 2022).

All of the negative consequences, which affected most of the essential components of the supply chain in the Ports of Los Angeles and Long Beach, obstructed the just-in-time service to the end consumers. Beyond its core and primary purpose, the JIT approach served as a goal for reaching sustainability. The availability of berths made it possible to reduce the ships' speed. It boosted the efficiency by determining the time of arrival and prevented the creation of the choke points at anchorages. The limited level of investments in port infrastructure and rail connections with main distribution centers resulted in the overall logistical chain disruption with the ports as the primary nodes.

Two methodologies used in calculating the air emissions of anchored ships in the area of Ports of Los Angeles and Long Beach sign up the significance of these data in the crisis period. The whole port system should build the port's resilience strategies and protocols to enhance reliability, sustainability, and efficiency. They should include the improvement in both foreland and hinterland connections providing the necessary data through an integrated information system which should prevent unannounced vessels callings to the ports and thus contribute to the environmental degradation and congestion. The severe impact of COVID-19 on the supply and demand balance and overall connecting activities chain in both port and traffic systems was a primary trigger for changing dynamics of the global economy and additional pollution in port cities. The decision-makers should include the sustainability parameter focusing on environmental protection as the highest priority if the determined initiatives of the EU, IMO, and other relevant institutions are reachable. It means the transition to alternative and renewable fuels, with green hydrogen, methanol, and ammonia, leading the industry to the zero-emission target. However, the radical change in energy sources and combustion engines certainly requires the reciprocity of significant investments in port infrastructure, equipment, and other relevant resources. If there is a congestion on the approach to the port, it is necessary to consider the reduction in ship's speed. According to the simulation presented, there are also significantly lower emissions on the examined route besides the realized fuel savings. Perhaps a "queuing on route" managed by the port operator could elegantly avoid queues in front of the port, and thus shifting the emissions outside the populated areas. Throughout the anchored ships' emissions calculation in the Ports of Los Angeles and Long Beach area, the authors faced several limitations, especially in setting up a comprehensive database and data availability. The crucial assumption was in the parameter of the average time of ships at anchor in ports. The retrieved dataset was available only for the Port of Los Angeles, so the same was used for the other port (LB). The authors also assumed that all anchored vessels inside or outside SAQA never used the main engine. It enabled the determination of the lower limit of air emission quantities. For the lack of official data, the auxiliary engine tier standard was based on the standards of the main engine retrieved from the official port documentation from the previous years. Also, a similar procedure was applied for the containerships' year of construction data, presuming that all vessels sailed in San Pedro Bay area were built before 2001. The recommendations for future work include the analysis of ports and hinterland emissions to provide the complete transport system emissions. Additionally, based on the determined quantities of various sources of emissions, the calculation of external costs would indicate the monetary value of the social and environmental damage and thus contribute to the importance of internalization measures for the external costs in transport.

## Data Availability

The datasets used and/or analysed during the current study are available from the corresponding author on reasonable request.

## Abbreviations

CO_2_: Carbon dioxide
SO_x_: Oxides of sulphur
NO_x_: Oxides of nitrogen
PM_10_: Particulate matter with a diameter of 10 microns or less
PM_2.5_: Particulate matter with a diameter of 2.5 microns or less
IMO: International Maritime Organization
GHG: Greenhouse gases
JIT: Just-in-time
LA: Los Angeles
LB: Long Beach
DPM: Diesel particulate matter
HC: Hydrocarbons
CO: Carbon monoxide
CO2e: Carbon dioxide equivalent
y–o-y: Year on year
TTW: Tank to wheel
WTT: Well to tank
US: United States
SAQA: Safety and air quality area
TEU: Twenty foot equivalent unit
kW: Kilowatt
LF: Load factor
MDO: Marine diesel oil
ECA: Emission control area
MGO: Marine gas oil
NO_2_: Nitrogen dioxide
CARB: California air resources board
g/kWh: Gram per kilowatt hour
t/day: Tons per day
EU: European Union
